# 

**Published:** 1858-11

**Authors:** 


### CONTENTS.

ORIGINAL COMMUNICATIONS— PAGE.

Remarks on the Condition of the System, between the Period of Overpowering Stimulation and Reaction. By Orrin Smith, M.D. ........ 549
Address, Introductory to the Sixteenth Annual Course of Lectures in Rush Medical College. By J. W. Freer, M.D. ........ 562
Bilious Fever with Torpor of Brain. By Drs. Stone and Dewey ........ 573
Ipecacuanha in Delirium Tremens. By Gerhard Paoli, M.D ........ 576
Abstract of Proceedings of the Aurora City Medical Association ........ 577
Report of Committee on Practical Medicine ........ 581

BOOK AND PAMPHLET NOTICES—

Lectures on the Principles and Practice of Physic. By Thomas Watson, M.D. With additions by Francis Condie, M.D ........ 597
On the Treatment of Aneurism by Compression, and by the Injection with the Perchloride of Iron. By John Reddy, M.D. ........ 598
The Physician's Visiting List, Diary and Book of Engagements for the Year 1859 ........ 599
Case of Chronic Hydrocephalus, treated by Injections of Iodine ........ 600

EDITORIAL—

The Medical Department of the University of Michigan and Preliminary Education ........ 601
Enquiry Answered ........ 606
Obituary ........ 507

